# The implementation of a community-based aerobic walking program for mild to moderate knee osteoarthritis: A knowledge translation randomized controlled trial: Part II: Clinical outcomes

**DOI:** 10.1186/1471-2458-12-1073

**Published:** 2012-12-12

**Authors:** Lucie Brosseau, George A Wells, Glen P Kenny, Robert Reid, Andreas Maetzel, Peter Tugwell, Maria Huijbregts, Carolyn McCullough, Gino De Angelis, Lily Chen

**Affiliations:** 1 Public Health, specialization in Epidemiology, University Research Chair, School of Rehabilitation Sciences, University of Ottawa, Ottawa, Canada; 2 Epidemiology and Biostatistics, Department of Epidemiology and Community Medicine, University of Ottawa, Ottawa, Canada; 3 School of Human Kinetics, Faculty of Health Sciences, University of Ottawa, Ottawa, Ontario, Canada; 4 University of Ottawa Heart Institute, Ottawa, Ontario, Canada; 5 University of Toronto, Toronto, Toronto, Canada; 6 Epidemiology, Chairman, Centre for Global Health, Institute of Population Health, University of Ottawa, Ottawa, Canada; 7 Baycrest Centre, Toronto, Canada; 8 Inter-Action Rehabilitation Inc., Toronto, Ontario, Canada; 9 Department of Epidemiology and Community Medicine, University of Ottawa, Ottawa, Canada; 10 University of Ottawa Heart Institute, Ottawa, Ontario, Canada

**Keywords:** Osteoarthritis, Clinical trial, Walking, Compliance, Adherence, Education, Behavioural intervention, Guidelines implementation, Knowledge translation

## Abstract

**Background:**

Osteoarthritis (OA) is the most common joint disorder in the world, as it is appears to be prevalent among 80% of individuals over the age of 75. Although physical activities such as walking have been scientifically proven to improve physical function and arthritic symptoms, individuals with OA tend to adopt a sedentary lifestyle. There is therefore a need to improve knowledge translation in order to influence individuals to adopt effective self-management interventions, such as an adapted walking program.

**Methods:**

A single-blind, randomized control trial was conducted. Subjects (n = 222) were randomized to one of three knowledge translation groups: 1) Walking and Behavioural intervention (WB) (18 males, 57 females) which included the supervised community-based aerobic walking program combined with a behavioural intervention and an educational pamphlet on the benefits of walking; 2) Walking intervention (W) (24 males, 57 females) wherein participants only received the supervised community-based aerobic walking program intervention and the educational pamphlet; 3) Self-directed control (C) (32 males, 52 females) wherein participants only received the educational pamphlet. One-way analyses of variance were used to test for differences in quality of life, adherence, confidence, and clinical outcomes among the study groups at each 3 month assessment during the 12-month intervention period and 6-month follow-up period.

**Results:**

The clinical and quality of life outcomes improved among participants in each of the three comparative groups. However, there were few statistically significant differences observed for quality of life and clinical outcomes at long-term measurements at 12-months end of intervention and at 6- months post intervention (18-month follow-up). Outcome results varied among the three groups.

**Conclusion:**

The three groups were equivalent when determining the effectiveness of knowledge uptake and improvements in quality of life and other clinical outcomes. OA can be managed through the implementation of a proven effective walking program in existing community-based walking clubs.

**Trial registration:**

Current Controlled Trials IRSCTNO9193542

## 

### 

Osteoarthritis (OA) is one of the most disabling degenerative diseases affecting the elderly [1]. While the reported prevalence of knee OA is approximately 3% among individuals between the ages of 45 to 54 years, this number rises to 27% for those between 63 and 69 years, and increases once again to 44% for persons above the age of 80 years [2]. The critical challenge is to develop physical activity **(PA)** programs that will encourage individuals with OA to not only to initiate, but also to maintain improvements in exercise behaviour over a long-term period. This study implemented relatively low-cost community-based walking programs at existing walking clubs.

The second part of this manuscript examines the effect of a proven effective walking program based on the Ottawa Panel CPG [3-5] and implemented through the use of a multifaceted KT intervention (Part I) [6]. This portion focuses on the “Outcome evaluation (Clinical outcomes)” phase of the Knowledge Translation Action Cycle **(KTAC)**[7,8] introduced in part I [6].

## Background

### Physical activity and quality of life

PA can exacerbate age-related decreases in joint health, functional status, and quality of life (QOL) [9-11]. Moreover, the prolonged physical inactivity observed in individuals suffering from arthritis increases their risk for chronic disease such as coronary heart disease, diabetes, hypertension, obesity and osteoporosis [12,13].

A recent study reported that only 27.8% of individuals with OA engaged in PA on a regular basis (i.e. 20 minutes a day, 5 days per week, or 30 minutes per day, 3 days per week) [13]. Promotion of PA, especially in a community-based context, is a priority for health organizations serving the general population [13,14] and is highly recommended for subgroups affected by chronic diseases including OA [15-17].

Various PA programs for OA have shown significant and beneficial effects on QOL at 2 and 3 months [18-24]. However, this effect was not maintained after a period of unsupervised PA [20,21,25]. According to Hurley [26], “exercise benefits people with OA while people are exercising; when they stop exercising the benefits can be maintained for a short time, but the gains are likely to be lost overtime unless individuals are actively encouraged to continue exercising”.

### Behavioural interventions

Behavioural interventions have been used with other chronic conditions to improve long-term maintenance of PA regimens with varying success. Patient education, health counselling, goal setting, telephone contacts, exercise logs, social/peer support and positive feedback either alone or in various combinations, have been studied in cardiovascular disease [27-30] and rheumatoid arthritis [31]. As with OA, multifaceted approaches appear to have the greatest impact on long-term adherence, degree of PA performed, and QOL.

The purpose of this randomized controlled trial **(RCT)** was to compare 1) improvements in quality of life **(QOL)** (primary outcome) and clinical outcomes such as pain, mobility and endurance; 2) adherence rates; and 3) confidence and self-efficacy after the implementation of a 12-month supervised community-based aerobic walking program (**SCAWP**) based on the Ottawa Panel clinical practice guidelines **(CPG**) [3-5] among three knowledge translation **(KT)** intervention arms. QOL, confidence, and self-efficacy were compared at 12- months (end of treatment) and at 18-months (6-months post-intervention). Adherence was compared during the intervention period at 3, 6, 9 and 12- months. Adherence, confidence and self-efficacy results are presented in part I of this manuscript.

## Methods

### Design

A single blind randomized controlled trail was used for this study. The hypothesis of this study was that a supervised community-based walking program would have a positive impact on participants with mild to moderate OA of the knee, if they walked regularly as stated in the recommendations of the Ottawa Panel [3-5]. This community-based study was approved by the University of Ottawa Research Ethics Board as well as by the City of Ottawa Public Health Research Ethics Board. All participants signed informed consent. Please see part I for more details on the study design [6].

### Sample and recruitment

A total of 222 adults with mild to moderate OA of the knee were recruited from Ottawa, Ontario and Gatineau, Québec, Canada. Please see part I for inclusion/exclusion criteria [6].

### Intervention

Participants were randomly assigned to one of the three KT intervention groups using central randomization [32] and computer generated numbers [33]: 1) Walking and Behavioural intervention **(WB)** ( implementation strategy) (18 males, 57 females) which included the SCAWP with a behavioural intervention and an educational pamphlet on the benefits of walking; 2) Walking intervention **(W)** (implementation strategy) (24 males, 57 females) wherein participants only received the SCAWP intervention and the educational pamphlet; 3) Self-directed control **(C)** (dissemination strategy) (32 males, 52 females) wherein participants only received the educational pamphlet. All 3 groups were provided with pedometers and log books to be completed to measure physical performance and additional PA aside from the walking sessions. The KT and dissemination strategies were implemented over a 12-month duration and participants were assessed for additional 6 months (15 and 18-month assessments). A detailed description of each intervention can be found in part I of this manuscript.

### Data collection

Participants were assessed by an independent evaluator at baseline and at each 3 month interval (months 3, 6, 9, 12) during the intervention period. Participants were then assessed at 3 and 6 months post-intervention during the follow-up period (months 15 and 18). Participants were asked to complete a collection of validated questionnaires as well as perform physical tests as each assessment.

### Clinical outcome measures

#### Health-related quality of life

The Arthritis Impact Measurement Scale (AIMS2) and the Short Form (SF-36) questionnaires were administered during patient evaluations. The scientific literature in rheumatology recommends that researchers use the AIMS2 and SF-36 concurrently, as they complement one another [34-36]. In support of their complementary roles, the detection of clinically important changes is enhanced through the use of a disease specific instrument (AIMS2), while a generic measure (SF-36) properly distinguishes different levels of self-reported general health statuses and co-morbidities not necessarily related to OA.

The SF-36 is the most widely used and extensively validated generic measure of health-related QOL [36-38]. The SF-36, which consists of 8 subscales and 36 questions, is a reliable and valid scale for several medical conditions, including rheumatic diseases [35,36,39-41]. Internal consistency via Cronbach’s alpha ranged between 0.74 to 0.96 over the different sections [40]. Test-retest intra-class correlation coefficients range from 0.75 to 0.93 [41]. Validity testing revealed that the SF-36 correlates moderately with several other measures related to disease activity, pain, depression and QOL [36,41]. The SF-36 was used to report QOL in several RCTs involving various PA programs designed for individuals with OA [19,21,24]. The responsiveness of this instrument was demonstrated in these studies by appropriate and expected changes in mean scores according to age, sex, marital status, severity of OA, and presence or absence of co morbid conditions. Mean scores also changed appropriately over time in response to the interventions. The aggregated SF-36 components are scored in which higher scores represented better health-related quality of life [42].

The Arthritis Impact Measurement Scale (AIMS2) is a disease-specific QOL scale [36] that consists of 101 items and includes various subscale scores [42]. The AIMS2 walking and bending sub-scale was considered as the primary outcome. All other AIMS2 sub-scales were used as secondary outcomes, since the use of the global scale could not be aggregated. The global AIMS2 scales are scored in which a low value indicated a higher health status [43,44].This QOL instrument has been used in several walking program studies that included subjects with OA [18,22,45]. AIMS2 has been shown to possess good internal consistency (alpha coefficients ranging from 0.72 to 0.91) and good test-retest results (Intraclass correlation coefficients ranging from 0.78 to 0.94). The construct and criterion validity have also been examined. Standardized response means for changes in AIMS2 scores over 3 months ranged from 0.36 to 0.80 [36,44].

Functional status was measured using the Western Ontario and McMaster Universities Osteoarthritis Index (WOMAC) (Bellamy and Buchanan, 1986). WOMAC is a self-administered multidimensional index containing dimensions for pain, stiffness and function. This five-point instrument has been shown to be valid and reliable in individuals with hip or knee rheumatic conditions, and is sensitive to change over time. The WOMAC questionnaire [45] has been adapted for lower extremity joints affected with OA, such as the knee or hip [19,22,45-48]. WOMAC is widely used and has been extensively validated [49,50]. Furthermore, according to Rogers & Irrgang [50], the WOMAC pain and functional subscales exhibit excellent responsiveness. The minimal clinically important rehabilitation effects ranged from 0.80 to 1.01 [51].

Other quantitative functional outcomes such as walking endurance and gait speed [20,22,52-55] were measured using the 6-minute walk test [55] and Timed-up-and-Go test [56].

#### Level of physical activity

Changes in level of PA were measured using the Seven Day Physical Activity Recall (7-day PAR) [57-59]. The 7-day PAR is a validated instrument in a calendar format in which a participant can indicate the duration (minutes per day), frequency (days per week) and intensity (multiples of basal metabolic rate). An adapted version of the 7-day PAR was included in the logbooks where PA was split into distinct walking and other physical activities.

### Analysis

A repeated measure mixed model was used to assess the change in health-related QOL measures (adjusted AIMS2 for age and co-morbidity and SF-36) as well as clinical outcomes from baseline to 12 months (end of intervention) and to 18 months (6 month follow-up post-intervention) among three study groups. The model included variables such as intervention group, study month, and an interaction term between the intervention group and study month. Missing data was assumed to be missing at random (MAR) in order to include incomplete data. Pair wise differences comparing each group to one another (W vs. C, W vs. WB, WB vs. C) were assessed. The repeated measure mixed model was used to compare the change of outcomes from baseline to the 18-month follow-up. Statistical analyses were performed using SAS (version 9.2, SAS Institute Inc., Cary, North Carolina), and statistical significance was defined as p < 0.05.

## Results

### Clinical outcome measures

#### Quality of Life

##### Short-Form 36

There were no statistically significant results between the three groups for QOL measured with the SF-36 (Table 1) with the exceptions of the variables “physical role functioning”, “physical role”, “pain index” and “standardized physical component”. At 12-months, results favoured the W group for the “physical role” variable when compared to the WB group (p = 0.04). At 18-months, results favoured the W group for the “physical functioning” (p = 0.02) and “pain index” (p = 0.00) variables when compared to the WB group. The self-directed group was also favoured when compared either to the W or WB groups for the “physical functioning” (p = 0.01), “pain index” (p = 0.03) and “standardized physical component” (p = 0.00) variables.

**Table 1:** Summary of SF-36 for three study arms

**Variables**	**Baseline**			**12 Months**			**18 Months**		
	**W**	**WB**	**C**	**W**	**WB**	**C**	**W**	**WB**	**C**
	**N**	**N**	**N**	**N**	**N**	**N**	**N**	**N**	**N**
	**Mean ± SD**	**Mean ± SD**	**Mean ± SD**	**Mean ± SD**	**Mean ± SD**	**Mean ± SD**	**Mean ± Sd**	**Mean ± SD**	**Mean ± SD**
				**W Vs. C(P)**	**WB Vs. C(P)**	**WB Vs. W(P)**	**W Vs. C(P)**	**WB Vs. C(P)**	**WB Vs. W(P)**
Physical Functioning	79	69	74	44	43	41	44	42	36
	63.003 ± 18.332	66.449 ± 19.157	67.275 ± 20.446	70.088 ± 18.819	68.127 ± 19.694	68.171 ± 26.381	68.157 ± 21.308	63.254 ± 25.705	75.694 ± 19.645
				(0.250)	(0.982)	(0.234)	(0.836)	*(0.012)	*(0.015)
Role Physical	78	69	74	44	44	41	44	42	36
	52.350 ± 40.064	69.203 ± 35.393	56.757 ± 39.755	61.742 ± 39.755	59.659 ± 41.138	65.854 ± 42.484	57.386 ± 40.556	60.119 ± 40.971	68.519 ± 39.345
				(0.909)	(0.063)	*(0.044)	(0.864)	(0.058)	(0.071)
Pain Index	78	69	74	44	44	41	44	42	36
	60.487 ± 16.040	67.348 ± 18.261	62.149 ± 18.762	63.818 ± 19.134	63.795 ± 20.124	67.805 ± 18.378	65.045 ± 18.881	61.167 ± 18.322	67.444 ± 18.315
				(0.581)	*(0.026)	(0.082)	(0.491)	*(0.030)	*(0.003)
General Health Perceptions	79	68	74	44	44	41	44	42	36
	67.873 ± 18.214	70.838 ± 20.465	70.635 ± 17.434	67.619 ± 17.484	68.926 ± 19.008	72.000 ± 18.358	69.307 ± 19.189	66.143 ± 20.483	69.917 ± 18.243
				(0.223)	(0.089)	(0.605)	(0.226)	(0.496)	(0.051)
Vitality	79	68	74	44	44	41	44	42	36
	58.481 ± 17.711	60.588 ± 21.152	60.473 ± 18.984	62.045 ± 19.628	60.341 ± 19.778	64.756 ± 18.267	60.152 ± 21.858	58.929 ± 18.791	66.157 ± 17.839
				(0.856)	(0.475)	(0.359)	(0.845)	(0.517)	(0.380)
Social Functioning	79	69	74	44	44	41	44	42	36
	80.380 ± 22.080	85.507 ± 18.394	82.432 ± 20.474	79.545 ± 22.889	84.375 ± 18.309	85.671 ± 20.264	78.125 ± 26.704	84.226 ± 19.533	79.167 ± 21.547
				(0.266)	(0.168)	(0.775)	(0.232)	(0.735)	(0.379)
Role Emotional	78	69	74	44	44	41	44	42	36
	75.214 ± 37.000	84.058 ± 29.488	74.324 ± 37.643	85.606 ± 30.835	81.061 ± 32.468	82.927 ± 35.057	75.000 ± 40.106	80.159 ± 31.286	82.407 ± 33.320
				(0.949)	(0.170)	(0.144)	(0.597)	(0.290)	(0.574)
Mental Health Index	78	68	74	44	44	41	44	42	36
	76.500 ± 17.558	77.588 ± 15.670	80.365 ± 13.974	78.364 ± 16.007	80.000 ± 14.823	81.366 ± 14.411	77.114 ± 17.926	77.405 ± 15.759	79.333 ± 14.890
				(0.735)	(0.909)	(0.822)	(0.597)	(0.880)	(0.486)
Health Transition Item	79	69	74	43	43	41	44	42	36 2.639 ± 0.899
	2.975 ± 0.987	3.014 ± 0.737	2.959 ± 0.818	2.419 ± 0.852	2.698 ± 0.914	2.463 ± 0.840	2.727 ± 0.924	3.024 ± 1.024	(0.459)
				(0.821)	(0.564)	(0.415)	(0.832)	(0.356)	
Standardized Physical Component	77	68	74	44	43	41	44	42	36
	40.516 ± 8.598	43.645 ± 8.656	41.996 ± 9.100	42.508 ± 9.229	42.192 ± 10.066	43.464 ± 9.409	42.820 ± 9.240	40.909 ± 11.038	45.149 ± 8.930
				(0.804)	(0.135)	(0.074)	(0.703)	*(0.009)	*(0.002)
Standardized Mental Component	77	68	74	44	43	41	44	42	36
	52.914 ± 10.845	53.812 ± 8.639	53.556 ± 8.995	53.819 ± 9.852	54.476 ± 7.329	55.162 ± 8.540	51.993 ± 11.000	53.922 ± 9.023	53.101 ± 9.914
				(0.595)	(0.436)	(0.795)	(0.688)	(0.638)	(0.937)

The selected results for SF-36 analysis were presented at baseline, at end of intervention (12 months), and at 6-months post intervention (18- month follow-up). The mean values of the 8 domains of the SF-36 for the three study groups are detailed in Table 1. After the 12-month intervention phase of the walking program, the self-directed group (C) had the highest mean (± SE) “physical role” score 65.85(±42.48 ). The WB group demonstrated the lowest mean pain index score 63.80 (±20.12). At 18-months, the self-directed group (C) demonstrated the highest mean scores for “physical functioning” 75.69(±19.65), “pain index” 67.44(±18.32), and “ standardized physical component” 45.15(±8.93). The WB group had the lowest mean scores for “physical functioning” 63.25(±25.71), “pain index” 61.17(±18.32), and “ standardized physical component” 40.91(±11.04).

##### Arthritis Impact Measurement Scale 2 (AIMS2)

The AIMS2 analyses were performed at baseline, at end of intervention (12- months), and at 6-months post intervention (18- month follow-up) (Table 2). At 12-months, statistical significance was observed when the WB group was compared to the self-directed group (C), mean results (± SE) favoured the self-directed group (C) for the “hand and finger” (p = 0.04), “social activity” (p = 0.01), “arthritis pain” (p = 0.02), “symptoms component” (p = 0.02)and “social interaction component” (p = 0.01) variables. At 12-months, statistical significance was only observed when the W group was compared to the self-directed group (C), mean results (± SE) favoured the self-directed group (C) for the “social activity” (p = 0.01) variable.

**Table 2:** Summary of AIMS2 for three study arms

**Variables**	**Baseline**			**12 Months**			**18 Months**		
	**W**	**WB**	**C**	**W**	**WB**	**C**	**W**	**WB**	**C**
	**N**	**N**	**N**	**N**	**N**	**N**	**N**	**N**	**N**
	**Mean ± SD**	**Mean ± SD**	**Mean ± SD**	**Mean ± SD**	**Mean ± SD**	**Mean ± SD**	**Mean ± Sd**	**Mean ± SD**	**Mean ± SD**
				**W Vs. C(P)**	**WB Vs. C(P)**	**WB Vs. W(P)**	**W Vs. C(P)**	**WB Vs. C(P)**	**WB Vs. W(P)**
Mobility	79	69	74	44	44	41	44	42	36
	1.281 ± 1.511	1.058 ± 1.621	1.169 ± 1.479	0.614 ± 1.017	0.773 ± 0.866	0.720 ± 1.090	0.815 ± 1.189	0.869 ± 1.215	0.486 ± 0.898
				(0.662)	(0.276)	(0.121)	(0.714)	(0.086)	(0.156)
Walking and Bending	79	69	74	44	44	41	44	42	36
	4.044 ± 2.047	3.513 ± 2.538	3.792 ± 2.494	3.364 ± 2.216	3.695 ± 2.248	3.085 ± 2.490	3.670 ± 2.323	3.702 ± 2.402	2.708 ± 2.112
				(0.900)	(0.081)	(0.094)	(0.656)	*(0.044)	(0.099)
Hand and Finger	79	68	74	44	44	41	44	42	36
	0.704 ± 1.061	0.647 ± 1.033	0.412 ± 0.904	0.602 ± 0.931	0.455 ± 0.689	0.744 ± 1.937	0.616 ± 1.195	0.693 ± 0.917	0.569 ± 1.196
				(0.127)	*(0.039)	(0.573)	(0.314)	(0.552)	(0.678)
Arm Function	79	68	74	44	44	41	43	42	36
	0.363 ± 0.795	0.368 ± 1.010	0.264 ± 0.642	0.295 ± 0.726	0.591 ± 1.448	0.220 ± 0.699	0.581 ± 1.170	0.405 ± 0.843	0.194 ± 0.511
				(0.451)	(0.253)	(0.673)	(0.175)	(0.635)	(0.369)
Self Care	79	69	74	44	44	41	43	42	36
	0.391 ± 1.632	0.045 ± 0.247	0.194 ± 1.179	0.114 ± 0.509	0.028 ± 0.132	0.091 ± 0.358	0.087 ± 0.572	0.104 ± 0.435	0.000 ± 0.000
				(0.801)	(0.726)	(0.541)	(0.541)	(0.409)	(0.147)
Household Tasks	79	69	74	44	44	41	44	42	36
	0.539 ± 1.093	0.272 ± 0.806	0.450 ± 1.549	0.341 ± 1.004	0.114 ± 0.279	0.274 ± 0.713	0.412 ± 0.933	0.565 ± 1.379	0.122 ± 0.468
				(0.924)	(0.879)	(0.802)	(0.679)	*(0.024)	(0.053)
Social Activity	79	69	74	44	43	41	44	42	36
	4.530 ± 1.723	4.449 ± 1.774	4.639 ± 2.050	4.727 ± 1.573	4.453 ± 1.886	3.732 ± 2.188	4.625 ± 1.840	4.720 ± 1.860	4.281 ± 2.141
				*(0.013)	*(0.006)	(0.767)	(0.944)	(0.233)	(0.187)
Support From Family	79	69	74	44	43	40	44	42	36
	1.907 ± 2.286	1.778 ± 1.938	2.204 ± 2.158	1.989 ± 2.269	2.253 ± 2.379	2.328 ± 2.356	1.932 ± 2.437	1.979 ± 2.052	2.743 ± 2.606
				(0.605)	(0.100)	(0.237)	(0.308)	(0.824)	(0.414)
Arthritis Pain	79	69	73	44	44	41	44 3.	42	35
	4.396 ± 1.907	3.813 ± 2.175	4.270 ± 2.426	3.494 ± 2.383	3.793 ± 2.285	3.494 ± 2.383	440 ± 2.409	3.642 ± 2.157	3.400 ± 2.228
				(0.523)	*(0.019)	(0.075)	(0.836)	*(0.030)	*(0.013)
Work	37	25	40	15	11	18	12	9	14
	2.095 ± 2.302	1.975 ± 2.193	1.828 ± 1.958	1.542 ± 1.828	0.625 ± 0.791	1.875 ± 1.561	2.188 ± 2.450	2.083 ± 1.849	1.830 ± 1.826
				(0.536)	(0.171)	(0.428)	(0.730)	(0.825)	(0.608)
Level of Tension	79	69	74	44	43	40	44	42	36
	3.327 ± 1.850	3.338 ± 1.900	3.030 ± 1.718	3.085 ± 1.692	3.078 ± 1.731	2.972 ± 1.897	3.313 ± 1.969	3.012 ± 1.799	2.815 ± 1.601
				(0.728)	(0.810)	(0.916)	(0.804)	(0.825)	(0.628)
Mood	79	69	74	44	42	40	44	42	36
	2.036 ± 1.548	1.937 ± 1.479	1.726 ± 1.168	1.795 ± 1.636	1.818 ± 1.324	1.478 ± 1.278	1.795 ± 1.909	1.893 ± 1.290	1.531 ± 1.301
				(0.686)	(0.224)	(0.397)	(0.622)	(0.449)	(0.194)
Satisfaction	79	69	74	43	44	41	44	42	36
	2.567 ± 1.580	2.746 ± 1.762	2.523 ± 1.458	2.113 ± 1.818	2.333 ± 1.728	2.127 ± 1.640	2.183 ± 2.080	2.657 ± 1.885	1.934 ± 1.570
				(0.529)	(0.800)	(0.704)	(0.599)	(0.305)	(0.108)
Health Perception	79	68	72	44	43	39	42	42	34
	3.890 ± 2.417	3.340 ± 2.159	3.572 ± 2.464	3.644 ± 2.369	3.495 ± 1.778	3.340 ± 1.877	3.340 ± 1.952	3.658 ± 2.310	3.438 ± 1.926
				(0.420)	(0.117)	(0.418)	(0.311)	(0.473)	(0.073)
Arthritis Impact	76	65	70	39	40	36	34	39	30
	3.191 ± 2.757	2.769 ± 2.259	2.929 ± 2.407	2.372 ± 1.809	2.500 ± 2.334	2.222 ± 1.962	1.985 ± 2.112	3.013 ± 2.512	2.250 ± 1.897
				(0.431)	(0.077)	(0.308)	(0.317)	(0.140)	*(0.011)
Physical Component	79	68	74	44	44	41	43	42	36
	1.224 ± 0.830	0.991 ± 0.777	1.047 ± 0.747	0.883 ± 0.845	0.943 ± 0.549	0.856 ± 0.771	1.039 ± 1.005	1.057 ± 0.753	0.676 ± 0.612
				(0.554)	(0.352)	(0.121)	(0.562)	*(0.011)	*(0.040)
Affect Component	79	69	74	44	42	40	44	42	36
	2.681 ± 1.570	2.637 ± 1.505	2.378 ± 1.344	2.440 ± 1.502	2.438 ± 1.436	2.225 ± 1.445	2.554 ± 1.806	2.452 ± 1.423	2.173 ± 1.368
				(0.937)	(0.690)	(0.623)	(0.934)	(0.835)	(0.762)
Symptoms Component	79	69	73	44	44	41	44	42	35
	4.372 ± 1.907	3.813 ± 2.175	4.270 ± 2.426	3.523 ± 2.360	3.793 ± 2.285	3.494 ± 2.383	3.440 ± 2.409	3.642 ± 2.157	3.400 ± 2.228
				(0.523)	*(0.019)	(0.075)	(0.836)	*(0.030)	*(0.013)
Social Interaction Component	79	69	74	44	43	40	44	42	36
	3.241 ± 1.578	3.114 ± 1.552	3.421 ± 1.614	3.358 ± 1.506	3.353 ± 1.698	3.033 ± 1.977	3.278 ± 1.906	3.350 ± 1.556	3.512 ± 1.965
				(0.081)	*(0.006)	(0.268)	(0.388)	(0.539)	(0.127)
Role Component	37	25	40	15	11	18	12	9	14
	2.095 ± 2.302	1.975 ± 2.193	1.828 ± 1.958	1.542 ± 1.828	0.625 ± 0.791	1.875 ± 1.561	2.188 ± 2.450	2.083 ± 1.849	1.830 ± 1.826
				(0.536)	(0.171)	(0.428)	(0.730)	(0.825)	(0.608)

At 18- months, when the WB group was compared to the self-directed group (C), mean results (± SE) favoured the self-directed group for the “walking and bending” (p = 0.044), “household tasks” (p = 0.02), “arthritis pain” (p = 0.03), “physical component” (p = 0.01), and “symptoms component” (p = 0.03) variables. When the W group was compared to the WB group, results favoured the W group for the “arthritis pain” (p = 0. 01), “arthritis impact” (p = 0. 01), “physical component” (p = 0.04), and “symptoms component” (p = 0. 01) variables.

At 12-months, the WB group demonstrated the highest mean scores for the “arthritis pain” 3.79 (±2.29) and “symptoms component” 3.79 (±2.29) variables, and the lowest score 0.46 (±0.69) for the “hand and finger” variable. The W group had the highest mean scores for the “social activity” 4.73 (±1.57) and “social interaction component” 3.36 (±1.51) variables. The self-directed group (C) had the lowest “symptoms component” score 3.94 (±2.38). At 18-months, the WB group demonstrated the highest mean scores for the “walking and bending” 3.70(±2.40), “household tasks” 0.57(±1.38), “arthritis pain” 3.64(±2.16), “physical component” 1.06(±0.75), and “symptoms component” 3.64(±2.16) variables. The self-directed group(C) had the lowest scores for the “walking and bending” 2.708(±2.11), “arthritis pain” 3.40(±2.23), “physical component” 0.68(±0.61), and “symptoms component” 3.40(±2.23) variables.

### Clinical outcomes

#### Functional status

At 12 and 18- months, improvements were observed in each three comparative groups as the overall item scores decreased when compared to baseline (Table 3). No statistical significance was demonstrated for total WOMAC scores at 12-months. At 18 months, the self-directed (C) group demonstrated the lowest mean (± SD) “pain” scores 23.50 (±17.78). The only statistically significant difference in total functional scores found between the 3 groups was at 18-months which favoured the W group when compared to the WB group (±16.74).

**Table 3:** Summary of WOMAC for three study arms

**Variables**	**Baseline**			**12 Months**			**18 Months**		
	**W**	**WB**	**C**	**W**	**WB**	**C**	**W**	**WB**	**C**
	**N**	**N**	**N**	**N**	**N**	**N**	**N**	**N**	**N**
	**Mean ± SD**	**Mean ± SD**	**Mean ± SD**	**Mean ± SD**	**Mean ± SD**	**Mean ± SD**	**Mean ± SD**	**Mean ± SD**	**Mean ± SD**
				**W Vs. C(P)**	**WB Vs. C(P)**	**WB Vs. W(P)**	**W Vs. C(P)**	**WB Vs. C(P)**	**WB Vs. W(P)**
Pain	79	69	73	43	42	41	43	42	35
	31.15 ± 14.29	26.81 ± 14.92	30.30 ± 16.47	24.65 ± 15.78	25.32 ± 15.98	25.00 ± 19.44	23.60 ± 15.09	26.16 ± 17.97	23.50 ± 17.78
				(0.572)	(0.238)	(0.522)	(0.863)	(0.157)	(0.096)
Stiffness	79	69	71	44	41	40	43	42	35
	38.90 ± 18.01	36.41 ± 18.90	39.08 ± 19.81	30.96 ± 22.31	30.79 ± 19.58	28.43 ± 20.41	29.94 ± 20.43	31.40 ± 20.75	27.14 ± 18.80
				(0.125)	(0.405)	(0.486)	(0.494)	(0.423)	(0.890)
Physical Function	79	68	72	44	38	40	43	42	35
	28.16 ± 15.41	27.65 ± 18.22	26.89 ± 16.34	24.48 ± 13.79	25.27 ± 15.70	25.06 ± 13.53	18.20 ± 14.63	24.15 ± 17.24	19.40 ± 17.08
				(0.672)	(0.903)	(0.763)	(0.582)	(0.381)	(0.140)
Total WOMAC Score	79	68	70	43	41	40	43	42	35
	29.70 ± 14.09	28.27 ± 16.42	28.95 ± 15.28	21.05 ± 13.62	23.60 ± 13.61	22.32 ± 17.77	20.30 ± 13.97	25.58 ± 16.60	20.90 ± 16.74
				(0.612)	(0.475)	(0.821)	(0.464)	(0.129)	*(0.019)

W: Walking only group; WB: Walking and Behavioural Group; C: Self-directed group (unsupervised/self-directed); N: number of subjects in each comparative group; SD: standard deviation; vs: versus; data is presented as mean (standard deviation); P: p-value (statistical significance); * Statistically significant; WOMAC: Western Ontario MacMaster Osteoarthritis Index.

#### Walking endurance

There were no statistically significant results for the 6-min walk test among the three groups after 12 and 18- months (Table 4). Distance (in meters) increased during the 6- minute walk test at both 12 and 18 months when compared to baseline among all three groups (Table 5). At 12- months, individuals in the W group had the highest score 524.86 (±106.52). The self-directed (C) group had the highest score 540.35 (±103.37) at 18 months.

**Table 4:** Summary of 6-minute walking for three study arms (6 Minute Walk Test)

	**Baseline**			**12 Months**			**18 Months**		
	**W**	**WB**	**C**	**W**	**WB**	**C**	**W**	**WB**	**C**
	**N**	**N**	**N**	**N**	**N**	**N**	**N**	**N**	**N**
	**Mean ± SD**	**Mean ± SD**	**Mean ± SD**	**Mean ± SD**	**Mean ± SD**	**Mean ± SD**	**Mean ± SD**	**Mean ± SD**	**Mean ± SD**
				**W Vs. C(P)**	**WB Vs. C(P)**	**WB Vs. W(P)**	**W Vs. C(P)**	**WB Vs. C(P)**	**WB Vs. W(P)**
6-min walk	79	68	74	44	41	40	42	39	35
	456.45 ± 87.62	475.27 ± 90.77	490.89 ± 99.30	524.86 ± 106.52	509.41 ± 82.43	520.52 ± 115.14	492.91 ± 86.95	500.15 ± 77.46	540.35 ± 103.37
				0.063	0.535	0.215	0.366	0.318	0.902

W: Walking only group; WB: Walking and Behavioural Group; C: Self-directed group (unsupervised/self-directed); N: number of subjects in each comparative group; SD: standard deviation; vs: versus; data is presented as mean (standard deviation); P: p-value (statistical significance); * Statistically significant; 6mwt: 6-min walk test (walking endurance).

#### Gait speed

There were no statistically significant results at both 12 months and 18-months for gait speed (Table 5). These results were similar to walking endurance, as gait speed was calculated using the 6 minute walk test. At 12 months, the W group had the highest score 1.458(±0.296). At 18-months the self-directed (C) group had the highest score 1.501 (±0.287).

**Table 5:** Summary of Gait Speed for three study arms

	**Baseline**			**12 Months**			**18 Months**		
	**W**	**WB**	**C**	**W**	**WB**	**H**	**W**	**WB**	**C**
	**N**	**N**	**N**	**N**	**N**	**N**	**N**	**N**	**N**
	**Mean ± SD**	**Mean ± SD**	**Mean ± SD**	**Mean ± SD**	**Mean ± SD**	**Mean ± SD**	**Mean ± SD**	**Mean ± SD**	**Mean ± SD**
				**W Vs. C(P)**	**WB Vs. C(P)**	**WB Vs. W(P)**	**W Vs. C(P)**	**WB Vs. C(P)**	**WB Vs. W(P)**
Gait Speed	79	68	74	44	41	40	42	39	35
	1.268 ± 0.243	1.320 ± 0.252	1.364 ± 0.276	1.458 ± 0.296	1.415 ± 0.229	1.446 ± 0.320	1.369 ± 0.242	1.389 ± 0.215	1.501 ± 0.287
				(0.063)	(0.535)	(0.215)	(0.366)	(0.318)	(0.902)

W: Walking only group; WB: Walking and Behavioural Group; C: Self-directed group (unsupervised/self-directed); N: number of subjects in each comparative group; SD: standard deviation; vs: versus; data is presented as mean (standard deviation); P: p-value (statistical significance); * Statistically significant.

#### Timed up and Go

There were no statistically significant results for the Timed up and Go among the three groups at 12 and 18- months (Table 6). At 12 and 18-months, Timed up and Go scores (time in seconds) decreased in all three groups when compared to baseline (Table 4). Timed up and Go scores were lowest for all 3 groups at 12 months when compared to 18 months. At 12 months, the WB group had the lowest score 8.10 (±1.54) compared to the other two groups. At 18 months, the self-directed (C) group had the lowest score 7.88 (±1.89).

**Table 6:** Summary of Timed Up and Go for three study arms

	**Baseline**			**12 Months**			**18 Months**		
	**W**	**WB**	**C**	**W**	**WB**	**C**	**W**	**WB**	**C**
	**N**	**N**	**N**	**N**	**N**	**N**	**N**	**N**	**N**
	**Mean ± SD**	**Mean ± SD**	**Mean ± SD**	**Mean ± SD**	**Mean ± SD**	**Mean ± SD**	**Mean ± SD**	**Mean ± SD**	**Mean ± SD**
				**W Vs. C(P)**	**WB Vs. C(P)**	**WB Vs. W(P)**	**W Vs. C(P)**	**WB Vs. C(P)**	**WB Vs. W(P)**
TUG	79	69	74	44	41	41	42	39	35
	9.04 ± 2.72	8.58 ± 2.45	8.34 ± 3.28	8.12 ± 2.44	8.10 ± 1.54	7.65 ± 1.79	8.41 ± 2.05	8.40 ± 1.36	7.88 ± 1.89
				(0.796)	(0.770)	(0.576)	(0.860)	(0.434)	(0.525)

W: Walking only group; WB: Walking and Behavioural Group; C: Self-directed group (unsupervised/self-directed); N: number of subjects in each comparative group; SD: standard deviation; vs: versus; data is presented as mean (standard deviation); p: p-value (statistical significance); * Statistically significant TUG: timed up and go.

#### Level of physical activity

After participating in the 12-month walking program [3-5], the WB group demonstrated the highest increase in activity level (Table 7). Statistical significance was not reached for “Leisure-Time Physical Activity” variables. At 12 months, the WB demonstrated the highest mean scores for “Leisure-Time Physical Activity Walking only” 13.89 (±12.40) and for “Leisure-Time Physical Activity Walking and other activities” 19.77 (±15.85). At 18 months, the self-directed (C) group demonstrated the highest mean score of 16.88 (±17.50) for “Leisure-Time Physical Activity Walking only” and 24.18 (±25.59) for “Leisure-Time Physical Activity Walking & other activities”. At 12 and 18 months, the WB group had the highest mean score for “Occupational/Domestic Activity Walking only” 33.00 (±70.75) and 33.07 (±39.04) and the highest “Occupational/Domestic Activity Walking and other activities” mean score 41.48 (±61.94). Statistical significance was demonstrated for “Occupational/Domestic Activity Walking and other activities” mean scores at 12- months 27.99 (±37.28) and 18 months 26.13 (±15.64) for the WB group.

**Table 7:** Summary of 7 day Physical Activity Recall for three study arms

**Variables**	**Baseline**			**12Months**			**18Month**		
	**W**	**WB**	**C**	**W**	**WB**	**C**	**W**	**WB**	**C**
	**N**	**N**	**N**	**N**	**N**	**N**	**N**	**N**	**N**
	**Mean ± SD**	**Mean ± SD**	**Mean ± SD**	**Mean ± SD**	**Mean ± SD**	**Mean ± SD**	**Mean ± SD**	**Mean ± SD**	**Mean ± SD**
				**W Vs. C(P)**	**WB Vs. C(P)**	**WB Vs. W(P)**	**W Vs. C(P)**	**WB Vs. C(P)**	**WB Vs. W(P)**
LTA Walking Only	63	49	56	42	37	38	40	33	32
	12.09 ± 13.09	13.92 ± 16.50	12.45 ± 14.96	12.22 ± 7.86	13.89 ± 12.40	12.68 ± 11.20	12.20 ± 9.90	16.40 ± 18.72	16.88 ± 17.50
				(0.816)	(0.816)	(0.987)	(0.261)	(0.932)	(0.303)
LTA Walking +Other	69	61	62	43	38	41	41	35	33
	16.18 ± 21.49	16.43 ± 18.86	19.74 ± 22.87	15.34 ± 10.23	19.77 ± 15.85	16.01 ± 14.14	16.46 ± 13.17	22.15 ± 21.21	24.18 ± 25.59
				(0.633)	(0.139)	(0.294)	(0.404)	(0.602)	(0.165)
ODA Walking Only	35	28	34	9	11	13	17	14	8
	20.75 ± 25.69	11.92 ± 12.42	18.17 ± 25.98	17.10 ± 21.03	33.00 ± 70.75	11.05 ± 8.39	22.33 ± 26.10	33.07 ± 39.04	12.04 ± 5.64
				(0.675)	*(0.040)	(0.113)	(0.601)	(0.076)	(0.140)
ODA Walking +Other	60	47	55	26	24	30	29	24	19
	31.31 ± 36.00	19.77 ± 25.61	26.85 ± 30.88	22.63 ± 20.97	41.48 ± 61.94	27.99 ± 37.28	23.34 ± 22.40	27.97 ± 33.15	26.13 ± 15.64
				(0.273)	*(0.047)	*(0.003)	(0.198)	(0.331)	*(0.021)

W :Walking only group; WB: Walking and Behavioural Group; C: Self-directed group (unsupervised/self-directed); N: number of subjects in each comparative group; SD: standard deviation; vs: versus; data is presented as mean (standard deviation); p: p-value (statistical significance); * Statistically significant; ODA: Other domestic activities; LTA: Leisure time activities.

## Discussion

We attempted to find the best way to implement a proven effective SCAWP from a clinical point of view [3-5]. As expected from the Ottawa Panel CPG on SCAWP [3-5], QOL as well as clinical outcomes of this RCT improved among participants with mild to moderate OA in each of the three comparative groups. However, very few statistically significant differences were observed for QOL and clinical outcomes over a long duration after the 12-month intervention phase and the 18-month follow-up phase. Results varied among the three groups depending on which outcomes were considered.

Participants from each group showed improvements in QOL and clinical outcomes measurements as they benefited from the walking component which has found be effective according to the Ottawa Panel CPGs [3-5]. The behavioural component facilitated the implementation of The Ottawa Panel CPG on SCAWP [3-5] over a short-term period (See part I), but did not have a direct impact on QOL and clinical outcomes [6].

### Quality of life (QOL) outcomes

Various PA programs, including walking programs for OA, have improved QOL when compared to a control group with no walking involvement over a short-term period [18-24]. However, this effect was not maintained after a period of unsupervised PA at 6 months [24], 9 months [20], and 18 months [25]. This RCT used a self-directed walking group as comparator.

Surprisingly, all statistically significant results favoured the self-directed group (C) group compared to the two walking groups (W and WB groups) with QOL improvements at 12 and 18 months. The behavioural component of the WB group was expected to improve participant adherence to the program over a long period of time, allowing participants to benefit from the proven effective walking program by improving and maintaining QOL and clinical outcomes. Some explanations as to why participants in the self-directed (C) group demonstrated a higher QOL score may be due to the development of fewer health problems and fewer difficulties with transportation resulting in less attrition to the study. In addition, participants in the self-directed (C) group may not have experienced as many barriers as the other two groups in order to engage in walking activities for at least 3 times a week. This group had the comfort of being around their own environment, especially for those who were not retired and still working. These facilitators may have contributed to the improvement of the participants’ QOL compared to the other groups which were required to travel regularly to the walking club.

Although the behavioural component of the WB group demonstrated the highest compliance rate for the first three months (Part I), they did not exhibit the highest QOL scores [6]. The self-directed (C) group contained the highest dropout rate, leading one to wonder if only participants with a higher QOL decided to adhere to regular walking.

### Clinical outcomes

The results of primary RCTs which served to develop Ottawa Panel CPGs [3-5] revealed that 10–60 minute aerobic walking programs for OA are proven effective to reduce pain intensity [18,22,47,52,53,60-62], to reduce morning stiffness [20] , to increase strength [55,63], to improve walking endurance [52,64] , to better manage stairs climbing [59,60,62], to increase the number of steps performed per day [62], to build self-efficacy on stairs and in walking [65], to improve functional status [24,47,59,64-66] and contribute to enhance QOL [20,24,47,52,67] when compared to a control group with no involvement of walking.

The nature of the walking program involved in the three comparative groups of this KT RCT demonstrated no differences between groups. The results of Evick et al. [47] revealed that a home-based exercise program is as effective as a supervised facility-based walking program for significantly improving pain relief, physical function, and quality of life at follow-up 3 months. Talbot et al. [55] observed that a home-based walking combined with education compared to education only improved the number of steps walked per day, improved the amount of time to climb stairs, and improved strength after the 12 week intervention and at follow-up 3- months post-intervention. The combined walking and education group also demonstrated improved pain relief at follow-up 3-months post intervention. Similar to a study by Minor et al. [20], the self-directed (C) group which was home-based, demonstrated the greatest improvements in clinical outcomes.

PA creates a motor evoked potential drop which decreases the corticomotor excitability which consequently reduces pain [68]. In a trial using rats, exercise has been shown to reverse signs of neuropathic pain and increase endogenous opioid content in brainstem regions which are important for pain modulation [69,70]. Several PA programs, including walking, have been shown to result in decreased pain in the lower extremities in those suffering from OA [18,20,22,47,53,67,71,72]. In these studies, the duration of the programs varied between 2 and 6 months. The study by Minor [20] demonstrated that the reduction in pain persisted after completion of the supervised aerobic walking program (3 and 9-month follow-up) if subjects continued to engage in PA. In this RCT, knee pain reduction, decreased morning stiffness and improved functional status were observed in all three comparative groups.

Surprisingly, there were no statistically significant results for “Leisure-Time Physical Activity” compared to “Occupational/Domestic Activity”. One reason for these results may be that long term goal setting, included in the Pace-ex program for those in the WB group, were not based solely on walking activities and included any daily activities (e.g. weight loss). The two other comparative groups did not contain a goal setting intervention. In addition, baseline results illustrated that participants in the self-directed (C) group received a higher level of education. Strong associations have been found between level of education, physical fitness level, and amount of leisure activity.

### Limitations

A major limitation of this study was the high attrition rate in each implementation and dissemination groups. As a result, validity of long-term results at 12-months end of intervention and at 18-months follow-up is questionable. Participants in the self-directed (C) group had initial lower values (poorer QOL) while participants in WB group demonstrated higher baseline values (higher QOL) for the physical components of the SF-36 and AIMS2 questionnaires. This situation may have lead to a potential bottom effect, creating larger room for QOL (SF-36) improvements for the self-directed (C) group and a potential ceiling effect for the WB group, resulting in smaller incremental improvements for QOL.

The use of the AIMS2 questionnaire among this specific population is debatable. The study subjects enrolled in this RCT were quite functional according to our inclusion criteria. They had to experience a low level degree of pain intensity and must have been able to walk for more than 20 minutes consecutively. AIMS2 is a QOL measurement designed for individuals with more severe physical conditions. This may have led to a potential ceiling effect on certain sub-scales such as the physical components.

Another limitation may have been the nature of the self-directed (C) group. The intervention among this group consisted solely of the use of a pamphlet on walking. As a result, the self-directed (C) group was not an inactive group, and walked regularly according to their self-reported logbook reports. We believe that the self-directed group (C) benefited just as much as the other two groups from the positive effects of walking.

Lastly, there were missing data from the 7-day PAR questionnaire as not all participants completed their logbooks and had difficulties remembering which activities were performed, resulting in a potential recall bias.

### Implications

This RCT was a long-term KT as well as compliance study which addressed questions of clinical and scientific importance. This study was designed to improve the understanding of efficacy of KT strategies to promote the adoption and maintenance of a community-based proven effective walking program for OA in order to improve the QOL of participants. Results of this study revealed that a community-based location or home-based walking program may be an effective strategy to manage OA of the knee.

## Conclusions

The three walking groups demonstrated globally equivalent results for the implementation of KT strategies to improve QOL and clinical outcomes over a long-term period (12 and 18-months).

## Abbreviations

OA: Osteoarthritis; QOL: Quality of life; RCT: Randomized controlled trial; SCAWP: Supervised community-based aerobic walking program; CPG: Clinical practice guideline; KT: Knowledge translation; KTAC: Knowledge Translation Action Cycle; EBCPG: Evidence-based clinical practice guidelines; CONSORT: Consolidated Standards of Reporting Trials; WB (group): Walking and behavioural intervention; W (group): Walking intervention; C (group): Self-directed control intervention; PA: Physical activity; PACE-ex: Program for Arthritis Control through Education and Exercise; AIMS2: Arthritis Impact Measurement Scale 2; SF-36: Short-Form 36 Health Survey; WOMAC: Western Ontario and McMaster Universities Osteoarthritis Index; MAR: Missing at random; CIHR: Canadian Institutes of Health Research.

## Competing interests

The authors declare that they have no competing interests.

## Authors’ contributions

Dr. LB is a Full Professor of Rehabilitation, an epidemiologist and the principal investigator of this study and primary author of this manuscript. Dr. GAW, the co-principal investigator of this study, is senior biostatistician and director, Cardiovascular Research Methods Centre at the University of Ottawa Heart Institute, and is a leading expert in the design and analysis of clinical trials. He provided assistance with the methodology and statistical analysis of the study. Dr. GPK is a Full Professor of Physiology at the University of Ottawa, and director of the university’s Human Performance and Environmental Medicine Research Laboratory and Professional Fitness and Lifestyle Consultant Certification Training program. He assisted with the methodology of the study. Dr. RR is a senior researcher at the University of Ottawa Heart Institute and provided experience in applying innovative behavioural approaches aimed at increasing PA in healthy and chronic diseased populations. Dr. AM is a health economist and assisted with the economic evaluation concept in the original proposal. Dr. PT is a rheumatologist, an epidemiologist and chief of the Cochrane Musculoskeletal Group. He has experience in conducting RCTs and meta-analyses. He provided assistance with OA outcome measures in the study. Dr. MH & Ms. CM developed the PACEex program and was in charge of training the physical activity specialist. Mr. GDA was the research coordinator of this study and assisted with the writing of this manuscript. Ms. LC is a biostatistician and performed the analyses of this study. All authors read and approved the final manuscript.

## Authors’ information

Lucie Brosseau (Ph.D.), School of Rehabilitation Sciences, Faculty of Health Sciences, University of Ottawa, 451 Smyth Road, Ottawa, Ontario, Canada. K1H 8M5.Telephone number: (613) 562–5800, ext. 8015; Fax number: (613) 562–5428; E-Mail: Lucie.Brosseau@uottawa.ca

## Pre-publication history

The pre-publication history for this paper can be accessed here:

http://www.biomedcentral.com/1471-2458/12/1073/prepub
